# Impact of cropping system diversification on productivity and resource use efficiencies of smallholder farmers in south-central Bangladesh: a multi-criteria analysis

**DOI:** 10.1007/s13593-022-00795-3

**Published:** 2022-08-05

**Authors:** Shah-Al Emran, Timothy J. Krupnik, Sreejith Aravindakshan, Virender Kumar, Cameron M. Pittelkow

**Affiliations:** 1 Department of Crop Sciences, University of Illinois at Urbana-Champaign, Urbana, IL USA; 2 Sustainable Impact Platform, International Rice Research Institute (IRRI), Los Baños, Laguna Philippines; 3 International Maize and Wheat improvement Center (CIMMYT), Sustainable Intensification Program, House 10/B, Road 53, Gulshan-2, Dhaka, Bangladesh; 4 Arunachal University of Studies (AUS), Knowledge City, NH52, Namsai, Arunachal Pradesh 792103 India; 5 Department of Plant Sciences, University of California, Davis, CA USA

**Keywords:** Cropping system diversification, Sustainability, Rice, Resource use efficiency, Multi-criteria, Smallholder, Economic profitability

## Abstract

Diversification of smallholder rice-based cropping systems has the potential to increase cropping system intensity and boost food security. However, impacts on resource use efficiencies (e.g., nutrients, energy, and labor) remain poorly understood, highlighting the need to quantify synergies and trade-offs among different sustainability indicators under on-farm conditions. In southern coastal Bangladesh, *aman* season rice is characterized by low inputs and low productivity. We evaluated the farm-level impacts of cropping system intensification (adding irrigated *boro* season rice) and diversification (adding chili, groundnut, mungbean, or lathyrus) on seven performance indicators (rice equivalent yield, energy efficiency, partial nitrogen productivity, partial potassium productivity, partial greenhouse gas footprint, benefit-cost ratio, and hired labor energy productivity) based on a comprehensive survey of 501 households. Indicators were combined into a multi-criteria performance index, and their scope for improvement was calculated by comparing an individual farmer’s performance to top-performing farmers (highest 20%). Results indicate that the baseline system (single-crop *aman* season rice) was the least productive, while double cropped systems increased rice equivalent yield 72–217%. Despite gains in productivity, higher cropping intensity reduced resource use efficiencies due to higher inputs of fertilizer and energy, which also increased production costs, particularly for *boro* season rice. However, trade-offs were smaller for diversified systems including legumes, largely owing to lower N fertilizer inputs. *Aman* season rice had the highest multi-criteria performance index, followed by systems with mungbean and lathyrus, indicating the latter are promising options to boost food production and profitability without compromising sustainability. Large gaps between individual and top-performing farmers existed for each indicator, suggesting significant scope for improvement. By targeting indicators contributing most to the multi-criteria performance index (partial nitrogen productivity, energy efficiency, hired labor energy productivity), results suggest further sustainability gains can be achieved through future field research studies focused on optimizing management within diversified systems.

**Supplementary Information:**

The online version contains supplementary material available at 10.1007/s13593-022-00795-3.

## Introduction

A vital challenge in the twenty-first century is to produce sufficient amounts of food while protecting both environmental quality and rural communities’ economic well-being (Foley et al. 2011; Springmann et al. 2018). It is estimated that global food and nonfood demand may increase by at least 60% between 2010 and 2050, and South Asian countries with high population densities and changing dietary patterns will need to double their crop production (Ladha et al. 2016; Tilman et al. 2011). Since the Green Revolution, agriculture has relied heavily on intensification and agrochemicals to increase yields (Tilman et al. 2002). While productivity dramatically increased, non-intended consequences of intensification have included environmental externalities, loss of natural resource quality, and declines in biodiversity (Robertson and Swinton, 2005; Tilman et al. 2002).

Agricultural intensification is the process of increasing crop productivity per unit area, which can either include higher yields for a single crop or higher system productivity by growing multiple crops per year on the same land (i.e., higher cropping intensity) (Pingali, 2012). In some of South Asia’s most productive agricultural systems, cropping system intensification has occurred through growing multiple cereal crops within a year, such as in the irrigated rice-wheat cropping systems of the Indo-Gangetic plain or triple rice cropping systems in Vietnam (Chen et al. 2011; Ladha et al. 2016). However, the intensification of cereal systems is often associated with higher inputs of nutrients, water, agrichemicals, labor, and energy (Pingali, 2012; Tilman et al. 2002), leading to major environmental concerns including high carbon footprints and low nutrient and energy use efficiencies (Kumar et al. 2018; Ladha et al. 2009; Tilman et al. 2011). Consequently, attention is now being directed toward developing cropping systems with more favorable effects on the environment, particularly through increasing the diversity of crops grown (Alam et al. 2017; Kremen et al. 2012).

Crop diversification represents a key pathway for improving sustainability, where multiple species or crop types are grown in rotation in different seasons within the same calendar year (Kar et al. 2004; Tamburini et al. 2020). Research has shown this can provide numerous benefits, including increased productivity (Gan et al. 2015), enhanced nitrogen use efficiency (Mhango et al. 2012), and lower carbon footprints (Yang et al. 2014), in addition to enhancing other ecosystem services (Tamburini et al. 2020). These criteria are, however, not necessarily the most crucial for smallholder farming systems struggling with poverty and limited resources. Changes in cropping systems that compromise economic profitability or farm-level efficiencies of labor, fertilizer, or energy are not desirable. Thus, research must account for a range of sustainability indicators and their potential synergies and trade-offs, ideally under representative constraints and conditions for smallholders. For rice-wheat and continuous rice cropping systems, experimental studies have a long history comparing alternative sequences of different cultivated species to examine diversification options (Alam et al. 2017; Assefa et al. 2021; Hufnagel et al. 2020; Ladha et al. 2016). Conversely, much less information is available from on-farm studies considering what crop sequences provide the best opportunities for increasing system performance across multiple indicators to balance the objectives of economic profitability, food production, and efficient resource use in smallholder systems.

A small country of just 143,570 square km, Bangladesh has one of the world’s most dense populations with 163 million people (World Bank, 2019). To feed the projected population of 185 million by 2030, Bangladesh will need to increase food production by 17% to achieve national food security (Timsina et al. 2018). This demand puts immense pressure on the country’s already limited arable land area. Further conversion of non-agricultural land to farming is unlikely; thus, cropping system intensification is suggested as the most feasible pathway for increasing total food production (Krupnik et al. 2017; Timsina et al. 2018). Since independence in 1971, the government of Bangladesh has supported land-use intensification, with an initial focus on rice which now covers 78% of total arable land (BBS, 2016). While this helped Bangladesh achieve self-sufficiency in rice production (Timsina et al. 2018), policymakers have long understood the negative effects of rice-based systems on water consumption, carbon and energy footprints, and loss of diversity in crops and wildlife; hence, the government proposed a crop diversification policy as early as 1989 (BDP, 2018; MOA, 1989).

**Fig. 1:**
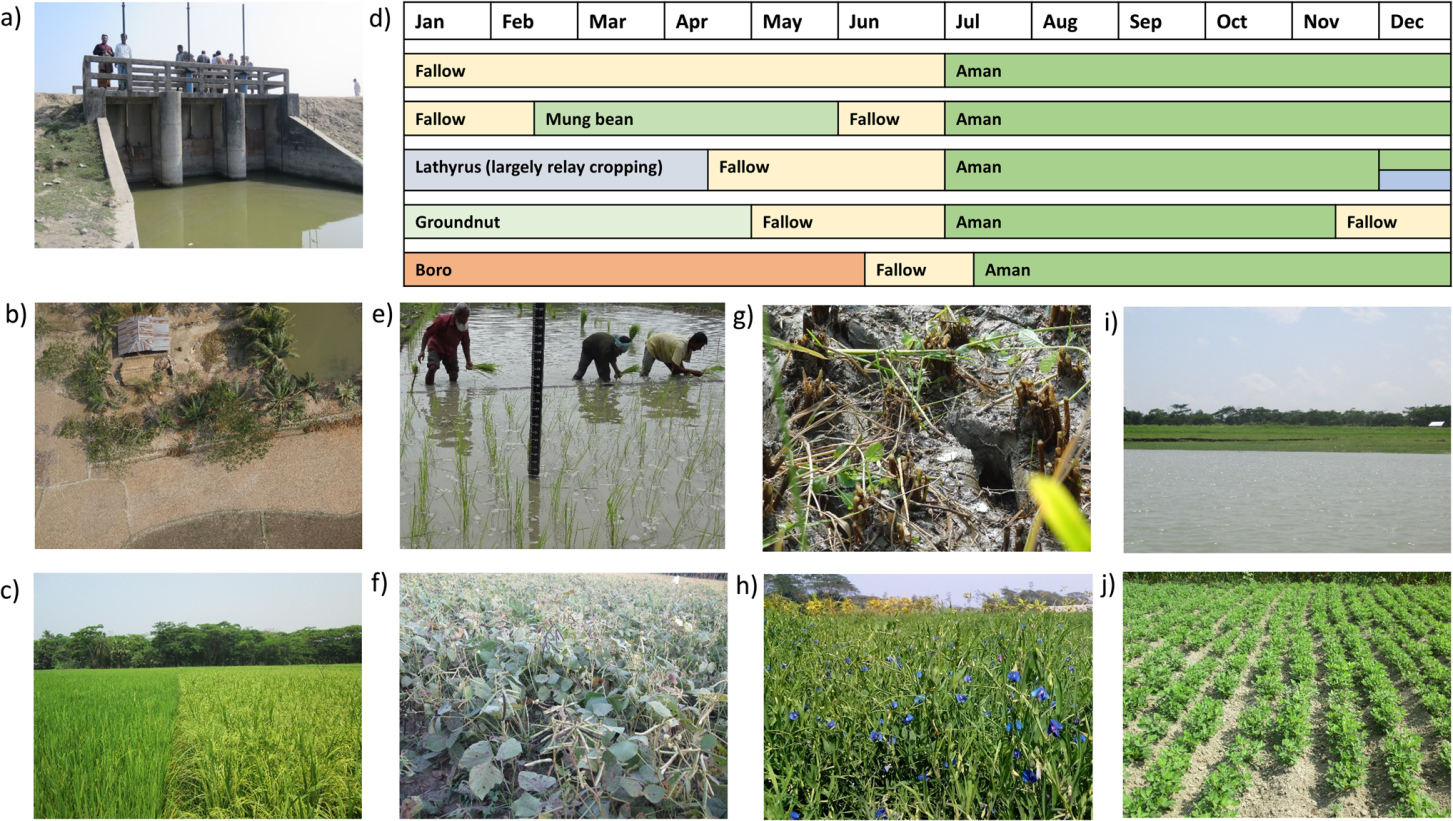
Images represent **a**) sluice gate and embankment of a polder, **b**) household with pond and fallow land after harvesting *aman* season rice, **c**) long-duration (left) and short-duration *aman* season rice, **d**) existing single and double cropped calender, **e**) Transplanting aman season rice in medium land (cm scale), **f**) mung bean (*Vigna radiate* L.), **g**) wet field during aman season rice harvesting, **h**) Lathyrus (*Lathyrus sativus* L.), **i**) land elevation (0–3 m), major river, and **j**) groundnut (*Arachis hypogaea* L.). Photographs by Shah-Al Emran.

Major crops grown in rotation with rice in Bangladesh are wheat, maize, potato, mustard, and winter vegetables using three cropping seasons (*boro*, *pre-monsoon*, and *monsoon*), but this is primarily in the northern and northeastern regions. In contrast, systems are less diversified in the Southcentral coastal zone, which consists primarily of one rice crop per year followed by a fallow season during the dry winter (*boro* season) (Krupnik et al. 2017). In this area, high and prolonged rainfall during the monsoon season (*aman* season), combined with the ebb and flow of tidal water movement into agricultural fields, forces farmers to rely on *aman* season rice as the cropping system base (Fig. 1). Three categories of *aman* season rice are cultivated depending on field landscape position and the corresponding level of water inundation a field experiences during the monsoon (i.e., flooding depth). Short-, medium-, and long-duration varieties are typically planted in high (0-–30-cm flooding depth), medium (medium-high and medium-low, 30–180 cm), and low (low and very low, >180 cm) landscape positions, respectively (Brammer, 2012; Emran et al. 2019). Due to climate risks and resource constraints (Aravindakshan et al. 2021), management of *aman* season rice is generally low input without irrigation, causing low average yields of 2.4 t ha^−1^ (BBS, 2016) with significant spatiotemporal variation (Assefa et al. 2021).

During the dry winter season, farmers have the potential to grow additional crops in coastal areas (Aravindakshan et al. 2021; Krupnik et al. 2017). Currently, the government is promoting irrigated rice (*boro* season rice) to overcome the low cropping system intensity compared to other regions. Farmers also cultivate groundnut, chili, mungbeans, and lathyrus, each having different input requirements and economic outcomes. However, we are not aware of research in Bangladesh quantifying how alternative crop sequences impact system performance in terms of agronomic, economic, and environmental indicators. Such holistic approaches are increasingly used to evaluate the sustainability of rice-based systems in other countries (Devkota et al. 2019; White et al. 2020). Optimizing outcomes across indicators is often challenging due to conflicts between food production and environmental goals, highlighting the need to explicitly assess trade-offs (Klapwijk et al. 2014). When considering multiple crops in rotation, research should account for the management practices and inputs associated with each crop relative to their outputs, which dictates system-level resource use efficiencies (Kumar et al. 2018; Ladha et al. 2016). For example, crops with lower inputs such as *aman* season rice may result in high resource use efficiency depending on yield levels, but whether this is also true for crops like mungbean and lathyrus remains unclear. On the other hand, irrigated rice grown during the *boro* season requires a higher amount of fertilizers (NPK), labor, and fuel for irrigation. While these inputs contribute to higher grain yield, production costs are higher, which may influence profitability and decrease resource use efficiencies.

In this study, we conducted a multi-criteria assessment of coastal smallholder cropping systems in southern Bangladesh to investigate opportunities for improving the dominant current system of low input, low productivity *aman* season rice. Using survey data from 501 households, our objectives were to (a) evaluate synergies and trade-offs of different cropping systems on farm-level productivity and resource use efficiencies, (b) develop a multi-criteria performance index using seven indicators considering agronomic, environmental, and economic dimensions, and (c) explore the scope for improving farm-level yield and resource use efficiencies for individual compared to top-performing farmers. Results show it is possible to increase productivity through higher cropping system intensity, but this comes with a cost of decreased resource use efficiencies and higher costs of production and climate risk, which can represent important trade-offs for smallholder farmers. However, crop diversification strategies with legumes resulted in a better balance of boosting food production without compromising environmental footprint, providing new insights for future research, extension, and policy in rice-based cropping systems.

## Materials and methods

### Study area

We studied existing smallholder cropping systems in south-central coastal Bangladesh, located in the active siltation zone of the Ganges-Brahmaputra estuary, consisting of many rives and tidal canals. Following previous work, our analysis focused on three districts—Patuakhali, Barguna, and Barishal (see the map of the study area in Aravindakshan et al. 2020 or Emran et al. 2021). The climate is subtropical and humid, with 1955 to 2100 mm annual rainfall. The majority soil type is silty clay loam, suitable for growing a wide range of crops. This area consists of hundreds of low-lying isolated islands (elevation 0–3m), separated by an extensive river and natural canal network. As a result, extreme weather events cause significant concerns for crop production in this area due to tidal flooding during the monsoon season, cyclones during May and November bringing high rainfall, and frequent drought during the dry season.

To support coastal farmlands against tidal flooding, cyclones, and saline water intrusion, Bangladesh’s government has developed polders, which are system embankments consisting of dykes and sluice gates to control water movement. Approximately 59 and 70% of households are involved in farming both outside and within polders, respectively (BBS, 2016). Polders are mostly developed in seventeen southern districts with closer proximity to the coastline, including Patuakhali and Barguna of south-central Bangladesh. In contrast, Barishal, the northernmost district of south-central Bangladesh, remains outside of polders, which makes it vulnerable to tidal flooding, but it generally does not experience soil or water salinity. While these three study sites are in the same agro-ecological zone, their different proximity to the coastline influences the crop choices due to differences in tidal flooding risks, drainage, soil salinity, and brackish water intrusion. Therefore, we evaluated cropping systems practiced by farming households within and outside of polders separately. For example, high tidal flooding can force farmers to cultivate long-duration traditional (local) rice varieties during the monsoon, which are only ready for harvest in late December or early January. Thus, it can be challenging for farmers to cultivate subsequent dry season crops on time, and fallowing their fields for half the year decreases cropping system diversity. While polders protect against tidal surges that can occur due to cyclones (Adnan et al. 2019; Sattar and Cheung, 2019), their construction changes the land and hydrology within polders, presenting other concerns. Water stagnation and land subsidence are key issues (Krupnik et al. 2015, 2017), reducing farmers’ options for diversifying their systems. Previous studies in the adjacent south-western coastal zone have observed a positive impact of polders on yield, economic performance, and cropping intensity (Chowdhury et al. 2010; Zaman and Mannaf, 2016), supporting our decision to separately analyze households within and outside of polders.

### Survey data and cropping systems evaluated

Comprehensive household survey data were collected through the Cereal System Initiative for South Asia (CSISA) project (Aravindakshan et al. 2020; http://hdl.handle.net/11529/10898), publicly available at the CIMMYT DataVerse. Please refer to Aravindakshan et al. (2020) for detailed information on the survey methodology and summary of household variables covering socioeconomic status, field characteristics, and crop management practices. The complete dataset includes agricultural livelihood information for 297 and 204 smallholder households located within and outside of polders, respectively, for the years 1995, 2000, 2005, 2010, and 2015. To address our research objectives with a focus on recent trends in farming systems and crop management, we used data from 2015 to estimate cropping system productivity, resource use efficiency, and environmental consequences, as described below. Variables used from the survey included information for land preparation, crops grown, agronomic inputs (fertilizer and pesticide use), total costs of production, labor use, and crop yield.

Cropping systems in this region are complex with the potential for species diversification depending on interactions between climate, hydrology, and available resources to invest in agricultural activities. There are three cropping seasons in Bangladesh:

- *Rabi* (typically mid-November to mid-March, also known as winter or *boro* season)
- *Kharif*-1 (typically mid-March to mid-July, also known as pre-monsoon or spring)
- *Kharif*-2 (typically mid-July to mid-November, also known as monsoon or *aman* season)

The *rabi* season is almost free from tidal flooding; this allows farmers to cultivate pulses, oilseeds, vegetables, and irrigated *boro* season rice. *Kharif*-2 is the most popular season for cultivating low input rainfed transplanted *aman* season rice. In between these two major cropping seasons, *kharif*-1 can be merged with *kharif*-2 or *rabi* crops, although much of the land in our study area remains fallow. During *kharif*-1, broadcast *aus* season rice was popular until the 1990s, but this system is no longer practiced in the study area.

In this study, we evaluated changes to the single-crop baseline system of *aman* season rice by considering additional crops grown during the *rabi* season. For the annual sequence of three seasons (*rabi*, *kharif*-1, *kharif*-2), survey results indicate that smallholders’ most typical cropping systems were fallow–fallow–*aman*, mungbean–fallow–*aman*, and lathyrus–fallow–*aman* (Table 1). Three minor systems included groundnut–fallow–*aman*, *boro*–fallow–*aman*, and chili–fallow–*aman*. As all survey participants cultivated *aman* rice during the *Kharif*-2 season, fallow-fallow-*aman* was considered as the control in this study. In 2015, no households cultivated *aus* season rice. Alternative crop sequences included both cropping system intensification (growing an additional *boro* season rice crop in *boro*-fallow-*aman*) and diversification (growing other non-rice species such as mungbean, lathyrus, groundnut, or chili after *aman* season rice). Because all cropping systems included *aman* season rice, just the additional *rabi* season crop is mentioned for simplicity when referencing each crop rotation in the text. Among 297 households within polders, 92.9 and 37.7% integrated mungbean and lathyrus into their systems, respectively, whereas 79.4 and 51.0% of the 204 households outside polders practiced these rotations. Less than 15% of households practiced the other cropping systems, including groundnut, *boro*, and chili. We did not include chili-fallow-aman and groundnut-fallow-aman cropping system in outside polders due to the small sample size (groundnut 2 and chili 7) (Table 1). Overall, cropping systems within polders showed relatively higher diversity than outside polders.

**Table 1:** Existing cropping systems identified in our survey and the extent to which they are practiced by farmers (number of households and % of total) in south-central Bangladesh. Due to small sample size, chili-fallow-*aman* and groundnut-fallow-*aman* were not included in further analysis for households outside polders.

Cropping system	Households within polders (% of total)	Households outside polders (% of total)
*Boro* – fallow – *aman*	11 (3.7)	17 (8.3)
Chili – fallow – *aman*	23 (7.7)	7 (3.4)
Fallow – fallow – *aman*	297 (100)	204 (100)
Groundnut – fallow – *aman*	44 (14.8)	2 (1)
Lathyrus – fallow – *aman*	112 (37.7)	104 (51.0)
Mungbean – fallow – *aman*	276 (92.9)	162 (79.4)

### Productivity and resource use efficiency indicators

For each cropping system, we calculated rice equivalent yield (Eq. 1) and total energy production (Eq. 3) as the primary indicators of productivity. This was necessary to standardize household production across the different crop sequences, including rice and non-rice crops. We did not consider economic productivity (gross margin) as a separate indicator because net economic returns are embedded in the rice equivalent yield formula below (Lal et al. 2017). All indicators were calculated on a per hectare basis covering a full annual production cycle for each cropping system (*rabi*, *kharif*-1, *kharif*-2).

1 $$ REY={Y}_x\times \frac{P_x}{P_r} $$

1a $$ \mathrm{Net}\ \mathrm{returns}=\mathrm{Gross}\ \mathrm{returns}-\mathrm{Cost}\ \mathrm{of}\ \mathrm{cultivation} $$

1b $$ \mathrm{Gross}\ \mathrm{returns}={Y}_x\times \mathrm{Unit}\ \mathrm{price}\ \left(\mathrm{BDT}\ {\mathrm{t}}^{-1}\right)\ast $$

1c $$ \mathrm{Cost}\ \mathrm{of}\ \mathrm{cultivation}={\sum}_{\mathrm{m}}^{\mathrm{i}}{\mathrm{Inputs}}_{\mathrm{i},\mathrm{m}}+{\sum}_{\mathrm{j}}^{\mathrm{i}}{\mathrm{Labor}}_{\mathrm{i},\mathrm{j}}+\mathrm{Land}\ \mathrm{preparation}+\mathrm{Irrigation} $$

Here, *REY*= rice equivalent yield (t ha^−1^ year^−1^) (*aman* season rice equivalent), *Y*_*x*_ = yield of the crop (t ha^−1^ Year^−1^) (for rice, *Y*_*x*_ = grain yield + grain equivalent straw yield), *Px* = price of the crop (BDT t^−1^), and *Pr* = price of *aman* rice (BDT t^−1^). In this calculation, rice price for each household was used to estimate rice equivalent yield, as selling prices differed from household to household. We considered straw values for rice gross return calculation. Units for net returns, gross returns, cost of cultivation, inputs, labor, land preparation, and irrigation were expressed in BDT ha^−1^ year^−1^.

Agronomic energy input (AEI, Equation 2, 2a, 2b), total energy production (TEP, Eq. 3), and net energy yield (NEY, Eq. 4) were calculated using standard energy conversion coefficients available in Supplementary Information (Table S1). Units for all energy inputs and outputs were GJ ha^−1^ year^−1^.

2 $$ AEI=\sum {\left({AEI}_a+{AEI}_{mi}\right)}_R+\sum {\left({AEI}_a+{AEI}_{mi}\right)}_{NR} $$

2a $$ {\mathrm{AEI}}_{\mathrm{a}}=\frac{I_a\times {EF}_a}{A} $$

2b $$ {AEI}_{mi}=T\ \mathsf{x}\ {EF}_{mi} $$

3 $$ TEP=\left(\mathrm{Aman}\  GY\ \mathsf{x}\ {EF}_{ry}\right)+\left(\mathrm{second}\ \mathrm{crop}\  GY\ \mathsf{x}\ {EF}_{gy}\right) $$

In Eqs. 2, 2a, 2b, *I*_*a*_ is the mass or volume of agronomic input *a* applied to the field with an area *A* (ha), *R* is *aman* season rice, NR is the non-*aman* season crop, EF_*a*_ is embedded energy for *a* (MJ kg^−1^ or MJ L^−1^), *T* is time (person-hours for labor) and EF_*mi*_ is the energy factor of human labor (MJh^−1^) (Table S1). In Eq. 3, GY indicates grain yield (t ha^−1^), EF_*ry*_ is the energy factor of the rice grain, and EF_*gy*_ is the energy factor of non-rice grain on an equivalent weight basis (Table S1).

Finally, net energy yield (GJ ha^−1^ year^−1^) was calculated as follows:

4 $$ NEY={TEP}_i-{AEI}_{ti} $$

Here, TEP_*i*_ is the annual total energy production of cropping system *i* calculated as in Eq. 3, and AEI_*ti*_ is the total agronomic energy input for the cropping system *i*.

Resource use efficiency indicators included energy efficiency, partial N productivity, partial K productivity, benefit-cost ratio, partial greenhouse gas (GHG) footprint, and hired labor energy productivity at the cropping system level calculated by the following equations:

5 $$ \mathrm{Energy}\ \mathrm{efficiency}=\frac{\mathrm{Net}\ \mathrm{energy}\ \mathrm{yield}}{\mathrm{Agronomic}\ \mathrm{energy}\ \mathrm{input}} $$

6 $$ \mathrm{Partial}\ \mathrm{N}\ \mathrm{productivity}=\frac{\mathrm{Total}\ \mathrm{energy}\ \mathrm{production}}{\mathrm{N}\ \mathrm{rate}\ } $$

7 $$ \mathrm{Partial}\ \mathrm{K}\ \mathrm{productivity}=\frac{\mathrm{Total}\ \mathrm{energy}\ \mathrm{production}}{{\mathrm{K}}_2\mathrm{O}\ \mathrm{rate}\ } $$

8 $$ \mathrm{Benefit}-\mathrm{cost}\ \mathrm{ratio}=\frac{\mathrm{Gross}\ \mathrm{returns}}{\mathrm{Total}\ \mathrm{cost}\ \mathrm{of}\ \mathrm{production}} $$

9 $$ \mathrm{Partial}\ \mathrm{GHG}\ \mathrm{footprint}=\frac{\mathrm{Partial}\ \mathrm{GHG}\ \mathrm{emissions}}{\mathrm{Total}\ \mathrm{energy}\ \mathrm{production}} $$

10 $$ \mathrm{Hired}\ \mathrm{labor}\ \mathrm{energy}\ \mathrm{productivity}=\frac{\mathrm{Total}\ \mathrm{energy}\ \mathrm{production}\ }{\mathrm{Hired}\ \mathrm{labor}\ } $$

Units for all energy inputs and outputs were expressed in GJ ha^−1^ year^−1^, partial GHG emissions (kg CO_2_ eq ha^−1^), total cost of production (USD ha^−1^ year^−1^, where 1 USD = 77.87 BDT), gross return (USD ha^−1^ year^−1^), and hired labor (person-days (PSD) ha^−1^). Economic performance was estimated as the benefit-cost ratio (Eq. 8) based on gross returns (Eq. 1b) and the total cost of production (Eq. 1c) considering all management operations, inputs, and hired labor.

In this study, only partial GHG emissions were calculated to estimate partial GHG footprint as kg CO_2_ eq per GJ energy production (Eq. 9). This included GHG emissions associated with agronomic inputs and their corresponding CO_2_ eq using standard coefficients (Table S1), consisting of CO_2_, N_2_O, and CH_4_ emissions associated with the production and use of chemical fertilizer and fuel. Following Emran et al. (2019), we did not include field GHG emissions due to data limitations. In flooded rice systems, field GHG emissions are primarily methane (CH_4_) and, to a smaller extent, nitrous oxide (N_2_O) emissions. In its simplest form, the established IPCC methodology for estimating CH_4_ emissions is based on an emission factor multiplied by the duration of field flooding. However, the duration of flooding was not a question in our survey and water management in coastal regions depends on multiple factors including monsoon rains, the length of the growing season for different rice varieties, and the land elevation of different fields influencing flooding depth. Therefore, to be conservative, we did not consider CH_4_ emissions resulting from anaerobic decomposition and soil-plant-floodwater processes. Because all cropping systems included one season of rice except *boro*-fallow-*aman*, this approach had limited effects on the indicator partial GHG emissions. That is, relative differences between cropping systems would remain the same if CH_4_ emissions were included, except for the system including *boro,* which had two rice seasons. However, we did estimate both direct and indirect N_2_O emissions based on fertilizer N inputs and their associated losses following established IPCC methodology (Intergovernmental Panel on Climate Change (IPCC), 2006).

For each indicator, the impact of cropping system diversification was evaluated by calculating the relative change in performance for each system compared to single *aman* season rice within each household (Eq. 11). This approach allowed us to assess synergies and trade-offs of alternative cropping systems, with a desirable improvement in indicators representing a synergy and undesirable effects representing a trade-off.

11 $$ \mathrm{Relative}\ \mathrm{Change}=\frac{\left(\mathrm{System}\ \mathrm{value}-\mathrm{aman}\ \mathrm{season}\ \mathrm{rice}\ \mathrm{value}\right)\times 100}{\ \mathrm{aman}\ \mathrm{season}\ \mathrm{rice}\ \mathrm{value}} $$

### Multi-criteria performance index

Factor analysis is a widely used method to develop a multi-criteria performance index in different disciplines, with increasing implementation to assess the sustainability of agricultural systems (Laurett et al. 2021; Valizadeh and Hayati, 2021). The benefit of using factor analysis is the ability to create latent (or unobserved) variables which represent correlations among multiple observed variables, thereby reducing the complexity of the dataset based on the statistical interdependency among key cropping system performance indicators (Pett et al. 2003). We calculated a multi-criteria performance index through factor analysis considering the following variables: rice equivalent yield, energy efficiency, partial N productivity, partial K productivity, partial GHG footprint, benefit-cost ratio, and hired labor energy productivity. Total energy production was not included for this calculation because most of the resource use efficiency indicators were based on total energy production already. This performance index was calculated separately for polder and non-polder households. The sample size, sample to variable ratio, and sampling adequacy were sufficient for factor analysis (Comrey, 1973; Pett et al. 2003). The analysis suitability of the survey data was assessed by the Kaiser-Meyer-Olkin (KMO) measure of sampling adequacy (MSA) test (overall MSA 0.73 and 0.71 for polder and non-polder areas, respectively). The extraction method was principal component analysis (PCA) involving a Varimax (orthogonal) rotation. As a single factor described the majority of variance in polders and non-polder households, we considered the first factor score as the multi-criteria performance index in our analysis. To conduct factor analysis, we used the R packages “psych,” “psychTools,” and “GPArotation” (R version 3.5.2).

### Scope for farm-level improvement

In this study, the scope of improving farm-level performance was estimated by comparing the mean of the top twentieth percentile of farmers to individual farmers for each indicator following Eq. 12. Using the mean of the top 20% accounted for smaller sample sizes in polder areas.

12 $$ \mathrm{Scope}=\frac{P_{fy}-{A}_{FY}}{A_{FY}}\times 100 $$

Here, *P*_*fy*_ = potential cropping system performance obtained by the mean of the top twentieth percentile of farmers and *A*_*FY*_ = household cropping system performance. Calculating the difference between individual and top-performing farmers is increasingly used to understand opportunities for closing yield and resource use efficiency gaps in rice systems (Stuart et al. 2016; Saito et al. 2021; White et al., 2020).

### Statistical analysis

Statistical analysis was performed using SAS (version 9.4) (SAS Institute, 2013). We used the “PROC GLM” command for a linear regression model for a one-way analysis of variance (ANOVA). We considered the least squares means (LSMEANS) because of the unbalanced dataset (unequal sample size for different cropping systems), helping ensure robustness of the analysis. The model was configured with cropping system as a fixed effect, treating households within and outside polders separately. Where the *F*-test indicated significance, means were separated at alpha=0.05 according to Tukey’s HSD. All confidence intervals presented in this study represent least squares means (LSMEANS) at the 95% level, weighted based on sample size. Data were evaluated to ensure the assumptions of normality and variance were met. All bar plots were created using the “ggplot2” package of R (R version 3.5.2) (R-Core-Team, 2020).

## Results

### Productivity and resource use efficiency indicators

Rice equivalent yield of *aman* season rice was 2.48 and 2.16 t ha^−1^ year^−1^, within polders and outside polders, respectively (Table 2). Cropping system intensification (two rice crops including *boro*) and diversification (*aman* season rice plus a non-rice crop of chili, groundnut, lathyrus, or mungbean) showed significant effects (*P*<0.001) for all performance indicators. Results for each indicator followed a similar pattern in both areas. In terms of food production, the system with chili (7.78 t ha^−1^ year^−1^) and *boro* (6.13 t ha^−1^ year^−1^) produced the highest rice equivalent yield within and outside polders, respectively. In polders, this was followed by systems with *boro*, groundnut, and mungbean, while mungbean–fallow–*aman* was the second highest outside of polders. All double cropped systems increased rice equivalent yield compared to *aman* season rice, but gains with lathyrus were lowest in both areas.

**Table 2:** Farm-level productivity and resource use efficiency indicators for different cropping systems within and outside polders of south-central Bangladesh (± 95% CI). Means followed by the same letter are not significantly different at 0.05 confidence level (letters in columns not separated by solid line indicate differences at alpha=0.05 according to the Tukey’s HSD; Significant code *** 0.001 ** 0.01 * 0.05).

	Rice equivalent yield (t ha^−1^ year^−1^)	Partial N productivity (GJ kg^−1^ N)	Partial K productivity (GJ kg^−1^ K_2_O)	Energy efficiency	Benefit-cost ratio	Partial GHG footprint (kg CO_2_ eq GJ^−1^)	Hired labor energy productivity (GJ PSD^−1^)
Within polders							
*Boro* – fallow – *aman*	6.18 (±0.35) B	1.03 (±0.21) C	2.52 (±0.61) C	4.67 (±1.25) C	1.17 (±0.15) D	19.34 (±0.90) A	0.68 (±0.09) ABC
Chili – fallow – *aman*	7.78 (±0.24) A	0.76 (±0.14) C	0.65 (±0.42) D	9.21 (±0.87) B	1.40 (±0.10) CD	15.82 (±0.62) B	0.61 (±0.06) BC
Fallow – fallow – *aman*	2.48 (±0.07) E	2.25 (±0.04) A	4.33 (±0.12) B	11.77 (±0.24) A	1.42 (±0.03) C	7.12 (±0.17) D	0.74 (±0.02) A
Groundnut – fallow – *aman*	6.15 (±0.18) B	2.30 (±0.10) A	3.00 (±0.30) C	7.92 (±0.63) B	1.44 (±0.08) C	6.87 (±0.45) D	0.73 (±0.04) AB
Lathyrus – fallow – *aman*	4.03 (±0.11) D	1.84 (±0.07) B	5.12 (±0.19) A	8.74 (±0.39) B	1.58 (±0.05) B	7.97 (±0.28) C	0.67 (±0.03) B
Mungbean – fallow – *aman*	4.80 (±0.07) C	1.89 (±0.04) B	4.98 (±0.12) A	8.91 (±0.25) B	1.78 (±0.03) A	7.97 (±0.18) C	0.60 (±0.02) C
*F-value*	823.21***	118.85***	113.7***	86.96***	67.63***	266.54***	30.49***
Outside polders							
*Boro* – fallow – *aman*	6.13 (±0.26) A	0.96 (±0.16) C	2.45 (±0.52) C	4.28 (±0.93) C	1.09 (±0.13) C	20.65 (±0.79) A	0.65 (±0.07) A
Fallow – fallow – *aman*	2.16 (±0.07) D	1.93 (±0.05) A	3.75 (±0.15) B	10.04 (±0.27) A	1.22 (±0.04) C	8.35 (±0.23) C	0.64 (±0.02) A
Lathyrus – fallow – *aman*	3.73 (±0.10) C	1.62 (±0.06) B	4.49 (±0.21) A	7.53 (±0.37) B	1.44 (±0.05) B	9.05 (±0.32) B	0.58 (±0.03) B
Mungbean – fallow – *aman*	4.55 (±0.08) B	1.64 (±0.05) B	4.31 (±0.17) A	7.66 (±0.30) B	1.64 (±0.04) A	9.18 (±0.25) B	0.52 (±0.02) C
*F-value*	772.85 ***	63.37***	26.44***	88.21***	82.31***	290.71***	22.2***

Despite greater productivity from growing an additional crop during the *rabi* season, resource use efficiencies were often reduced with double cropped systems, particularly partial GHG footprint. Rice intensification with *boro* showed the lowest energy efficiency, benefit-cost ratio, and partial nitrogen use efficiency both within and outside polders, while also showing the lowest partial potassium efficiency outside polders. In addition, the *boro* system showed the highest partial GHG footprint. In contrast, diversification through the addition of legumes (mungbean, lathyrus, and groundnut) improved multiple performance indicators, particularly nitrogen and potassium use efficiency, profitability, and partial GHG footprint in comparison with the *boro* systems. In both areas, the integration of mungbean supported higher economic profitability with moderate efficiencies but low labor productivity. Groundnut integration had the best labor productivity, partial N productivity, and partial GHG footprint. However, the single-crop system of *aman* season rice still had among the highest nitrogen use efficiency, energy efficiency, and labor productivity, resulting from low inputs in this system. Chili was the only non-rice, non-legume crop considered in polder areas. The chili system produced the highest rice equivalent yield but had relatively poor partial N productivity, partial K productivity, benefit-cost ratio, partial GHG footprint, and labor productivity.

**Fig. 2:**
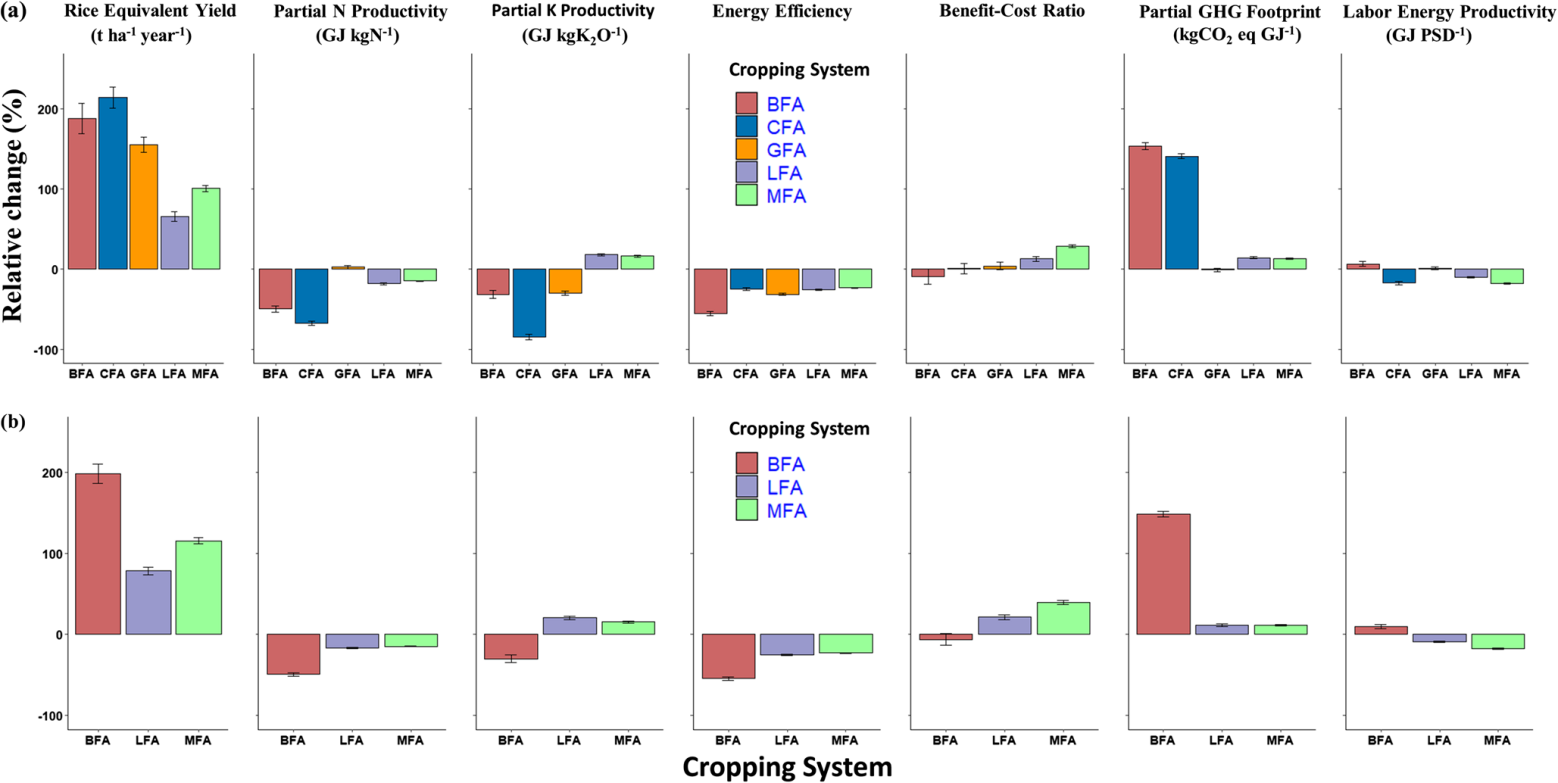
Farm-level synergies and trade-offs of different cropping systems compared to the baseline single *aman* season rice in (a) within polder and (b**)** outside polder. Values are expressed as percentage change relative to fallow-fallow-*aman* (± 95% CI) within (a) and outside (b) polders in south-central Bangladesh. Cropping system abbreviations are fallow–fallow–*aman* (FFA), groundnut–fallow–*aman* (GFA), *boro*–fallow–*aman* (BFA), chili–fallow–*aman* (CFA), mungbean–fallow–*aman* (MFA), and lathyrus–fallow–*aman* (LFA).

When indicators were expressed as percentage change compared to *aman* season rice, both synergies and trade-offs were observed with double cropping (Fig. 2). Double cropping increased rice equivalent yield by a range of 72–217%, but the magnitude differed for each system, with legumes showing lower increases than *boro* or chili systems. In both areas, double cropping had trade-offs in energy efficiency, partial nitrogen productivity, and partial GHG footprint in all systems except groundnut within polders. The chili and *boro* systems showed the highest trade-offs in partial nitrogen use efficiency within and outside polders, respectively, while *boro* showed the highest trade-offs across partial GHG footprint and other resource use efficiencies. Meanwhile, systems with mungbean and lathyrus decreased labor productivity but also reduced trade-offs while maintaining or increasing economic profitability in both areas, as did groundnut in polder areas.

### Multi-criteria performance index

**Fig. 3:**
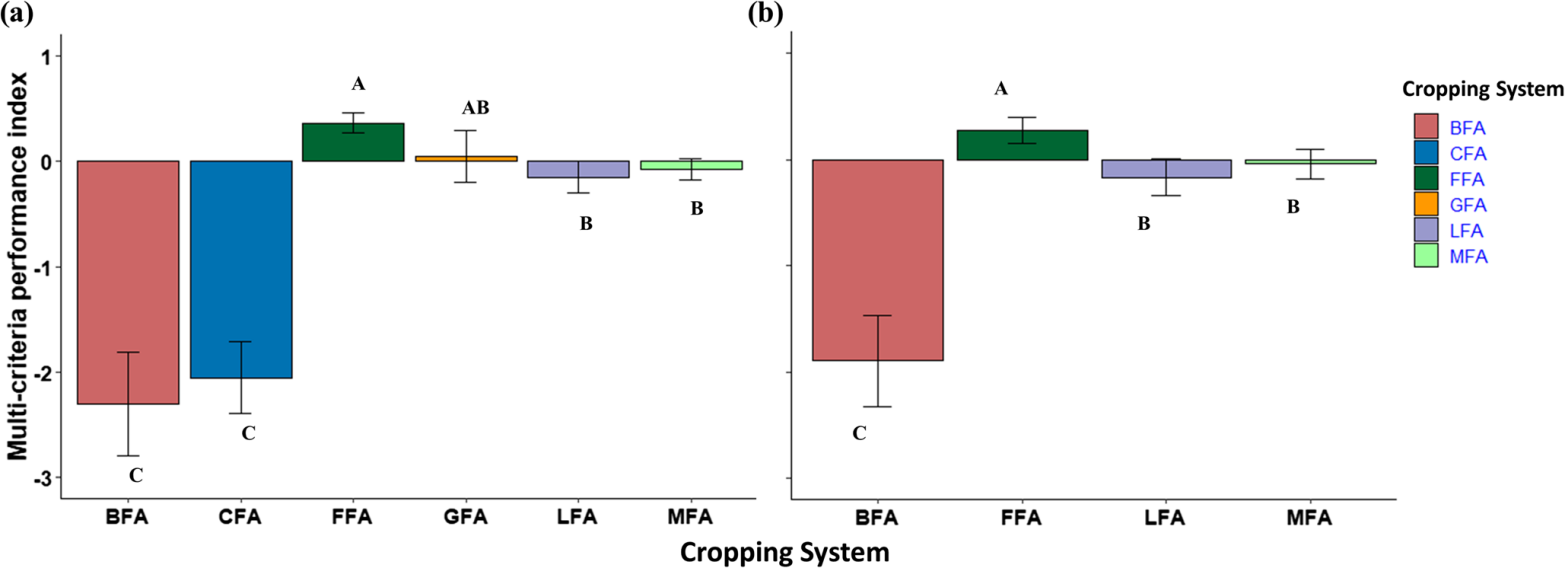
Multi-criteria performance index of different cropping systems( a) within polders and( b) outside polders in south-central Bangladesh. Means (± 95% CI) followed by the same letter are not significantly different at 0.05 confidence level. Cropping system abbreviations are fallow–fallow–*aman* (FFA), groundnut–fallow–*aman* (GFA), *boro*–fallow–*aman* (BFA), chili–fallow–*aman* (CFA), mungbean–fallow–*aman* (MFA), and lathyrus–fallow–*aman* (LFA).

The multi-criteria performance index represented 55 and 57% of variance in polder and non-polder households, respectively (Table 3; Fig. 3). Higher loading scores represent more influential variables that can positively or negatively affect the index based on loading dimension. Given that most variables represented efficiencies, higher values indicate improvements in sustainability. Hence, systems with a positive multi-criteria performance index, in this case, are more desirable, while systems with a negative index indicate lower food production and efficiencies, making them less desirable. In both polder and non-polder areas, *aman* season rice had the highest index, along with groundnut systems within polders. Other double cropped systems reduced the multi-criteria performance index, with the intensified *boro* system having the lowest value in both areas. Considering diversification options, cultivating legumes (mungbean, lathyrus, and groundnut) showed a higher multi-criteria performance index than chili within polders, and the *boro* system both within and outside polders.

**Table 3:** Factor loading for the 7 performance indicators are presented in addition to sum of squared (SS) loadings, proportion of variation explained, measure of sampling accuracy (MSA), and root mean square of residuals (RMSR).

	Factor loading	
	Within polder	Outside polder
Variables		
Rice equivalent yield (t ha^−1^)	−0.083	0.020
Partial N productivity (GJ kg N^−1^)	0.922	0.948
Partial K productivity (GJ kg K_2_O^−1^)	0.732	0.791
Energy efficiency	0.839	0.922
Benefit-cost ratio	0.596	0.622
Partial GHG footprint (kg CO_2_ eq GJ^−1^)	−0.853	−0.790
Hired labor energy productivity (GJ PSD^−1^)	0.81	0.796
Statistical Analysis		
Sum of squared (SS) loading	3.84	4.02
Proportion of variation	0.55	0.57
Overall MSA	0.73	0.71
RMSR	0.16; 0.19	0.20; 0.25

In both areas, partial N productivity had the biggest positive influence (loading scores of 0.922 and 0.948 in polder and non-polder, respectively) on the multi-criteria performance index followed by energy efficiency (0.84, 0.92), hired labor energy productivity (0.81, 0.80), partial K productivity (0.73, 0.79), and benefit-cost ratio (0.60, 0.62). In contrast to the productivity and efficiency indicators, the negative loading of partial GHG footprint reflects how this indicator corresponds with a negative environmental impact. That is, an increase in GHG emissions per unit of energy production decreased the multi-criteria performance index.

### Scope to improve farm-level performance

**Fig. 4:**
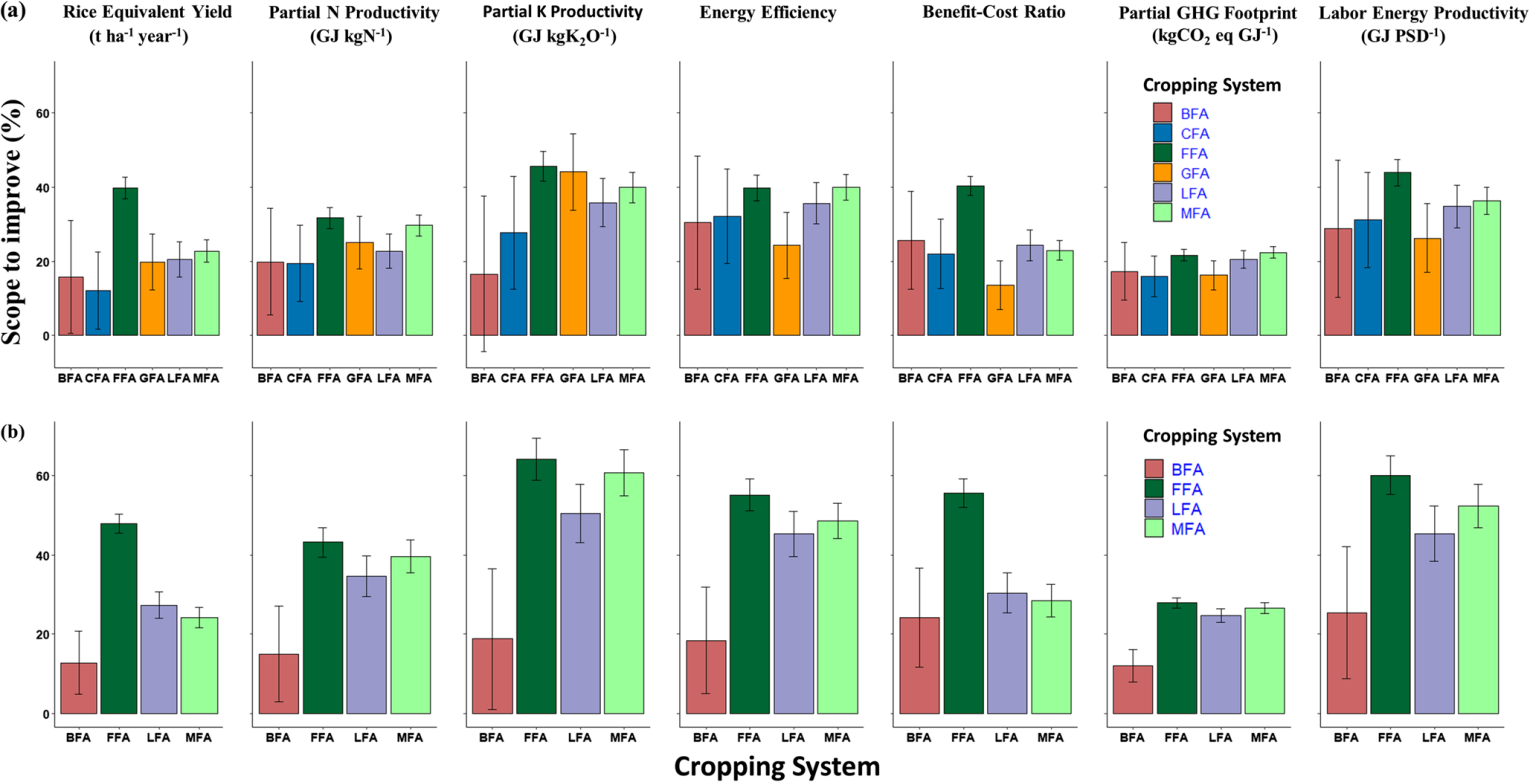
Scope to improve farm-level performance and minimize trade-offs for different cropping systems (a) within polders and (b) outside polders in south-central Bangladesh. Values were calculated as the difference between the top-performing farmers (mean of top 20 percent) and individual farmers for each indicator and system (± 95% CI). Cropping system abbreviations are fallow–fallow–*aman* (FFA), groundnut–fallow–*aman* (GFA), *boro*–fallow–*aman* (BFA), chili–fallow–*aman* (CFA), mungbean–fallow–*aman* (MFA), and lathyrus–fallow–*aman* (LFA).

Assessing values for each farmer compared to top-performing farmers revealed considerable scope to improve productivity, profitability, and resource use efficiency in all cropping systems (Fig. 4). In both areas, *aman* season rice had the largest room for improvement within each indicator, ranging from 32 to 43% for partial N productivity and 46–64% for partial K productivity. Among double cropped systems outside polders, those with *mungbean* and *lathyrus* showed higher scope to improve performance, except for economic profitability. Within polders, systems with chili and groundnut had the least overall room for improvement. Partial nitrogen use efficiency, which was earlier identified as the most important variable to increase the multi-criteria performance index, had room to increase by 15–42% depending on the cropping system. Similar trends were also observed for other performance indicators, where legume integration (mungbean, groundnut, lathyrus) resulted in higher (on average 30%) scope to improve performance compared to other double cropped systems, both within and outside polders. Overall, farmers outside polders showed similar or higher scope to improve performance compared to within polders.

## Discussion

### Limits of rice intensification

The low productivity of *aman* season rice favors intensification of this cropping system, because *aman* season rice alone cannot promise food security for Bangladesh’s growing population (Timsina et al. 2018). *Aman* season rice was the single most important crop cultivated by all farmers in this study, despite it being the least productive system (Table 2). Our findings are in line with Assefa et al. (2021) who reported the rice-fallow system was the least productive inside polders in southwestern Bangladesh. However, this system likely remains popular among farmers because it required the lowest amount of fertilizers (31–32 kg N ha^−1^), production costs (<430 USD ha^−1^), and energy inputs (6 GJ ha^−1^) (Table S2), which enhanced resource use efficiencies and placed *aman* season rice as the highest multi-criteria performance index (Fig. 3). Despite the sustainability benefits of this single crop system for the indicators evaluated here, increasing food production through cropping system intensification is a priority for coastal Bangladesh. Recent studies have shown the potential to cultivate two to three crops annually, producing 13–31 t ha^−1^ rice equivalent grain (Alam et al., 2015, 2017; Ladha et al. 2016). Similarly, Bhattacharya et al. (2019) showed that cropping system intensification with improved varieties and best management practices inside polders can achieve an annual productivity of 14–20 t ha^−1^ and gross income of USD 1200 ha^−1^ year^−1^.

An important question facing coastal regions is whether intensification should occur through adding an additional rice crop, grown under irrigation during the *boro* season (dry winter period), or through rotation with non-rice crops. Our results show that farmers practicing the intensified double rice system (*boro*-fallow-*aman*) can produce 3.70 and 3.97 t ha^−1^ more grain compared to *aman* season rice alone within and outside polders, respectively. To reduce the strain on groundwater resources in the north of Bangladesh, current government policy is emphasizing the need to increase cropping system intensity by expanding irrigated *boro* rice in coastal regions, replacing the current practice of fallowing land during winter (BDP, 2018; MoA, FAO, 2013). However, in our study, the double rice system required the highest labor, fertilizers, energy, and production costs (1355–1368 USD ha^−1^), which resulted in the lowest multi-criteria performance index. Moreover, cultivating two rice crops per year, particularly with high agrochemical use in the *boro* season, resulted in the highest GHG emissions per unit of energy input. At the national level, *boro* season rice has the greatest total water consumption and grey water footprint, an indicator of pollution, despite it occupying lower cropped area than *aman* season rice, contributing to significant concerns about the sustainability of water resources (Mullick and Das, 2021). These findings underscore the cropping system intensification challenges facing Bangladesh, where prominent trade-offs exist between food production and environmental goals, particularly related to water use and GHG emissions for rice production (Pandey et al. 2020; Sapkota et al. 2021).

Besides environmental footprint, the prospect for resource-poor smallholder farmers adopting input-intensive systems such as *boro* rice with low profitability and high production costs is extremely challenging in Bangladesh. Recent choice experiments with farmers illustrate there is little preference for *boro* rice as an intensification option compared to other crops (Aravindakshan et al. 2021), with *boro*-fallow-*aman* practiced by less than 10% of farmers in our study area. Indeed, *boro* rice area has been declining in the past decades, largely due to high fertilizer input and irrigation costs. Although the government recognizes the need to reduce reliance on *boro* rice for maintaining self-sufficiency in rice production, it still accounts for over 50% of national rice production. In this context, alternative cropping systems that meet food production and economic goals while reducing negative environmental externalities are urgently needed. Our results show that double cropping with higher diversity is an important tool to enhance rice equivalent yield and multi-criteria performance across different indicators.

### Overcoming trade-offs through diversification

Our findings support efforts to increase cropping system diversity, while highlighting the importance of different crop species and how alternative rotation sequences impact the required inputs for cultivation. Diversification through legumes (mungbean, groundnut, and lathyrus) boosted total system productivity while reducing trade-offs in resource use efficiency for double cropped systems, contributing to enhanced multi-criteria performance (Fig. 3). Assefa et al. (2021) also observed higher rice equivalent yield and economic performance in rice-mungbean systems compared to rice-fallow systems inside polders within 100 km of our study area. In contrast, although integration of chili achieved the highest rice equivalent yield within polders, diversification with this non-legume crop also reduced resource use efficiencies and increased GHG emissions. Legume integration into cereal-based cropping systems has been shown to provide multiple benefits, including increased profitability with decreased nitrogen, water, and GHG footprints (Alam et al. 2017; Hossain et al. 2016; Ladha et al. 2016). However, there are important aspects of risk and crop establishment to consider in coastal environments that may present challenges to smallholder adoption, discussed below.

The inclusion of legume crops in rice monoculture systems could increase soil fertility through biological nitrogen fixation (Bhuiyan, 2004), with estimates that cultivating mungbeans could add 25–40 kg N ha^−1^ to the soil-plant system (Ahlawat et al. 1998; Ali, 1992). Lowering the need for N fertilizer inputs is key for decreasing energy consumption, GHG emissions, and production costs. In our study, farmers applied 11–17 kg N ha^−1^ when growing mungbean, lathyrus, or groundnut, in addition to the N rate for *aman* season rice (31–32 kg N ha^−1^), whereas growing chili and *boro* required 60–120 kg N ha^–1^ extra N fertilizer (Table S2). Besides, mungbean and lathyrus required significantly less labor, K fertilizer, and production cost than *boro* season rice in both polder and non-polder households. The system with *boro* rice had the highest N inputs (150–153 kg N ha^−1^), causing the greatest trade-offs in resource use efficiencies, particularly partial GHG footprint as discussed above. These findings align with our recent field experiments in south-central Bangladesh, where increasing N rates for rice showed a clear trade-off between agronomic, economic, and environmental goals (Emran et al. 2019).

Several factors influence the potential for diversification of this baseline system. The successful integration of non-rice crops is largely controlled by climate and precipitation patterns, including field flooding due to high rainfall, the risk of tropical cyclones, soil and irrigation water salinity during the winter period, and poor drainage which can result in late planting (Assefa et al. 2021; Krupnik et al. 2015, 2017). Modern short-duration *aman* season rice varieties may have the highest scope for diversification because early harvesting of rice facilitates optimum planting time for high-value winter crops such as groundnut, chili, tomato, wheat, lentil, sesame, mustard, and other vegetables. For medium-duration *aman* rice varieties, farmers generally miss the early planting of winter crops due to a later rice harvest and wet soil, and therefore grow mungbean, lathyrus, or black gram as a second crop or leave land fallow. Finally, long-duration *aman* season rice varieties span almost both the monsoon and winter season, forcing farmers to grow lathyrus or leave fields fallow. Considering that about 80% of land in this region belongs to the medium land type (BARC, 2018), our results suggest that mungbean and lathyrus represent important crops that could be grown after medium-duration *aman* rice varieties to increase agricultural productivity and sustainability for smallholders. Importantly, these crops are already grown by a large proportion of farmers in this area (Table 1) and represent the best multi-criteria performance index for diversified systems.

### Significance of multi-criteria approach

To achieve agricultural sustainability, multi-criteria assessments are necessary to minimize trade-offs and identify cropping systems capable of promoting synergies between input use efficiencies, economics, and environmental costs. In this field of research, an important knowledge gap is not only considering positive and negative relationships among indicators but how to compare the overall performance of different cropping systems based on an integrated understanding of these relationships as a standard value. In our study, *aman* season rice had the highest sustainability, followed by cropping systems with mungbean and lathyrus among diversified systems, and lastly the system including *boro* rice (Fig. 3; Table 3). Previous work has shown the benefits of calculating a multi-criteria performance index as a composite value (Fadul-Pacheco et al. 2013; Sabiha et al. 2016). This is a step further than other research efforts focused on displaying synergies and trade-offs through visualization tools to inform policy (Kanter et al. 2018; Kumar et al. 2018), where the net impacts across multiple indicators are not always clear.

Consistent with our multi-criteria performance index, all farmers in this region practice the baseline system of *aman* season rice. The low input requirements of this system resulted in the highest nitrogen use efficiency, energy efficiency, and labor productivity, making it attractive to smallholders who are risk averse, despite it having the lowest productivity. Recent work in southwestern coastal areas also found that rice-fallow has a lower risk compared to other more profitable rotations such as rice-maize or rice-sunflower in polders (Assefa et al., 2021). Regarding the multi-criteria index, a novel insight from our study is that the economic gains from mungbean or lathyrus more than offset the lower labor productivity and marginal resource use efficiencies of these systems, helping them balance competing objectives across multiple dimensions of sustainability important to smallholders. In line with this result, recent work has shown that farmers prefer mungbean over maize or wheat as an intensification option, highlighting the need for improved mungbean varieties adapted to local conditions (Aravindakshan et al. 2021). While our results show clear benefits from diversification with legumes, we note that our index does not include aspects of required investment (capital costs of production) and risk (climate extremes causing unfavorable growing conditions) that are crucial elements of adoption for resource-limited smallholders.

Among the alternative crops evaluated, mungbean and lathyrus currently cover the most land area compared to higher-yielding and high-value chili, groundnut, maize, and other crops (BBS, 2016; Kamal et al. 2020). As noted above, reasons for farmers avoiding high-yielding dry season crops are the late harvest of *aman* rice, high soil moisture until mid-February, and lack of dry season irrigation access (Islam et al. 2016; Krupnik et al. 2017; Ritu and Mondal, 2004). Although the system with lathyrus shared a similar multi-criteria performance index as mungbean-fallow-*aman*, the latter contributed to a higher benefit-cost ratio (1.64 and 1.78; non-polder and polder) and grain yield, which may explain why mungbean is cultivated by more farmers in this region (Table 1). Besides, mungbean is also best suited in the high and medium highland covering more than 80% of this region’s cropland, as it can be planted during late winter. Lathyrus is also flexible and can be cultivated on different land types (based on flooding depth), solely as independent or relay crops. Lathyrus is a better second crop following long-duration *aman* season rice, particularly in medium-low and low land fields. Though groundnut-fallow-*aman* had a similar multi-criteria performance to systems with lathyrus and mungbean within polders, groundnut is more susceptible to waterlogging conditions and this physiological barrier limits where it can be planted. As groundnut grows better in sandy or silty loam soil, mostly found in this region’s highlands, it is not widely planted in flood-prone fields outside of polders (BARC, 2018; BBS, 2016). Therefore, expanding groundnut in medium highland fields which experience excess water will require developing improved varieties with waterlogging resistance.

### Scope for farm-level improvement

Potential gains in crop productivity or efficiencies are increasingly quantified in on-farm research by exploring variability among a population of farmers (Stuart et al. 2016; Saito et al. 2021). With this approach, the benchmark for current management is the performance of individual farmers, and the upper threshold of potential achievement is represented by top-performing farmers, which are assumed to be facing similar biophysical and socioeconomic constraints. In our study, we found significant scope for improving cropping system performance based on the combination of variation in environment and management practices across different indicators (Fig. 4). This difference between individual and top-performing farmers represents the gap in yield or efficiency that can theoretically be closed through improved agronomic management for a given indicator (Devkota et al. 2019).

Depending on the cropping system, we observed a 12–48% scope to improve rice equivalent yield, which can also be considered as the attainable yield gap (Fig. 4). This observation is in line with a 19–64% yield gap reported for different crops in other studies, including rice in Bangladesh (Assefa et al. 2021; Guilpart et al. 2017; Timsina et al. 2018). There are two other important points from our analysis of variability among farmers. First, *aman* season rice always had the largest gap between average and top-performing farmers across different indicators. This is because *aman* season rice is grown in all field types (i.e., different landscape positions), often with different varieties and varying amounts of inputs. Other studies have reported large spatiotemporal variation for rice yield in smallholder systems of Bangladesh, even under the same varieties and fertilizer inputs (Ara et al. 2017; Assefa et al. 2021). In southern Bangladesh, variation in soil fertility (mostly nitrogen) and field elevation (which controls flooding depth) are important determinants of rice yield (Ara et al. 2017; Ran et al. 2018). Because *aman* season rice is both the most widely practiced system and has the largest scope for improvement, our results suggest that developing and implementing improved management practices should remain a key priority for government research and extension programs.

Second, this analysis demonstrates the potential for closing resource use efficiency gaps in diversified cropping systems, which could reduce trade-offs and further increase the multi-criteria performance index. The results above show that systems with mungbean, lathyrus, and groundnut increased rice equivalent yield, yet higher inputs led to lower resource use efficiencies compared to *aman* season rice. Specific indicators to target for improvement are those contributing most to the multi-criteria index, including partial N productivity, energy efficiency, and hired labor energy productivity, all with loading scores of 0.80 or above. Across the different cropping systems, our analysis suggests that farmers can achieve large gains in partial nitrogen use efficiency (15–42%), energy efficiency (18–55%), and labor productivity (25–60%) by implementing management practices similar to the top-performing farmers.

A limitation of the survey data used in this study is that it lacked detailed agronomic information, preventing an analysis of differences in management for top farmers. Therefore, although our research represents an important step in integrating multiple dimensions of sustainability based on a combination of current diversification options and farmer practices, further agronomic field trials are necessary. Building on the work of others (Alam et al. 2015, 2017; Bhattacharya et al. 2019; Kumar et al. 2018; Ladha et al. 2016), such research should optimize management practices within diversified systems to increase partial nitrogen productivity, energy efficiency, and labor productivity, ideally under on-farm smallholder conditions. While our study focused on agronomic factors, socioeconomic constraints can play an equal or greater role regarding the potential for increasing cropping system intensity in smallholder systems. See Emran et al. (2021) for an in-depth discussion of how off-farm employment, farm size, distance to roads and markets, and access to capital, labor, inputs, and extension services all influenced farm-level household productivity.

## Conclusion

Bangladesh faces challenges regarding food security, land availability, and diminishing natural resources that will grow in the future due to increasing population and climate change. In this study, we evaluated a range of cropping systems currently practiced by smallholder farmers in the south-central coastal region to quantify gains in food production and economic profitability through intensification (double rice) or diversification (rice and non-rice crops), while simultaneously accounting for changes in resource use efficiencies leading to negative environmental impacts. Results show that the baseline single-crop system of *aman* season rice had the lowest productivity and economic performance, but it was also low input with low risk, translating into important sustainability benefits and the highest multi-criteria performance index. In contrast, double cropping with rice or other crops clearly improved total system productivity, but this also required additional inputs (fertilizers, labor, and energy), which increased production costs and caused a trade-off in environmental performance through lower resource use efficiencies. Among the double cropped systems, diversification through mungbean or lathyrus had lower trade-offs because these systems required the lowest fertilizer inputs and production costs. Finally, the large variability among farmers suggested that top-performing farmers have been able to implement management approaches and techniques to increase yield and mitigate these trade-offs, despite biophysical and socioeconomic challenges. We conclude that targeting improvements in key indicators (partial nitrogen productivity, energy efficiency, and hired labor energy productivity) through future field trials with mungbean and lathyrus could strongly affect the multi-criteria performance of diversified systems. Overall our results support diversification as a key principle of sustainable cropping system intensification, but government policy, research, and agricultural education programs are needed to further optimize management and address economic risk as a barrier to adoption. Of particular importance is that timely planting of winter crops following *aman* season rice, even short-duration varieties, remains a major challenge for enhancing diversification in low-lying coastal zones because high soil moisture in medium land fields hampers tillage, seed germination, and crop establishment. Smallholders face other large socioeconomic and environmental constraints, including climate shocks and limited access to inputs, credit, labor, roads, and markets. These broader challenges emphasize the need for holistic support systems and the co-creation of knowledge with farmers, researchers, and government institutions.

## Supplementary Information

ESM 1 (DOCX 23 kb)

## Data Availability

Data are publicly available at the CIMMYT DataVerse (http://hdl.handle.net/11529/10898).
