# Supplementary material for: Impact of cropping system diversification on productivity and resource use efficiencies of smallholder farmers in south-central Bangladesh: a multi-criteria analysis
Source: Agron Sustain Dev. 2022 Aug 5;42(4):78. doi: 10.1007/s13593-022-00795-3 (PMC9355929; doi:10.1007/s13593-022-00795-3)
Supplement: Supplementary file 1 — (DOCX 23 kb) [file 13593_2022_795_MOESM1_ESM.docx]

**Supplementary Information**

**Impact of cropping system diversification on productivity and resource use efficiencies of smallholder farmers in south-central Bangladesh: a multi-criteria analysis**

Submitted to Agronomy for Sustainable Development

Shah-Al Emran^*^, Timothy J. Krupnik, Sreejith Aravindakshan, Virender Kumar, and Cameron M. Pittelkow

^*^ Corresponding author. E-mail: [emran2@illinois.edu](mailto:emran2@illinois.edu)

Department of Crop Sciences, University of Illinois at Urbana-Champaign, Urbana, Illinois, United States of America

**Table S1.** Standard coefficients used in this study for calculating energy efficiency parameters and GHG emissions based on agricultural inputs and labor for each system. (^1^Values are obtained and converted from Lal (2004); ^2^Values are obtained and converted from IPCC (2006)).

| a) Energy conversion factors used in this study. | | | | | | | | |
| --- | --- | --- | --- | --- | --- | --- | --- | --- |
|  | | | | | | | | |
|  |  | | MJ unit^-1^ | | Source | | | |
| Material inputs | Diesel (L) | | 47.70 | | Pimentel and Pimentel, 2008; Quilty et al., 2014 | | | |
|  | Nitrogen (N kg) | | 66.14 | | Rahman and Hasan, 2014 | | | |
|  | Phosphorus (P_2_O_5_ kg) | | 12.44 | | Rahman and Hasan, 2014 | | | |
|  | Potassium (K_2_O kg) | | 11.15 | | Rahman and Hasan, 2014 | | | |
|  | Seed (kg) | | 15.50 | | Pimentel and Pimentel, 2008 | | | |
|  |  | |  | |  | |  | |
| Human labor | 2-wheel tractor operator (h) | | 0.98 | | Ainsworth et al., 2011; Quilty et al., 2014 | | | |
|  | Hired labor (h) | | 0.98 | | Ainsworth et al., 2011; Quilty et al., 2014 | | | |
|  |  | |  | |  | |  | |
| Seed, Outputs | Rice (kg) | | 15.20 | | Pimentel and Pimentel, 2008 | | | |
|  | Mungbean (kg) | | 14.7 | | Lal et al., 2015 | | | |
|  | Lathyrus (kg) | | 14.7 | | Lal et al., 2015 | | | |
|  | Groundnut (kg) | | 25 | | Nikkhah et al., 2015 | | | |
|  | Chili (kg) | | 0.8 | | Kuswardhani et al., 2013 | | | |
|  | | | | | | | | |
| b) Coefficients for greenhouse gas (GHG) emissions of agricultural inputs | | | | | | | | |
|  | | | | | | | | |
| Emission source | |  | | GHG | | Emission coefficients | | Unit |
| Production, transportation and storage of fertilizers ^1^ | | Nitrogen (N) | | CO_2_ | | 4.77 | | Kg CO_2_e kg^-1^ N |
|  |  | Phosphorus (P_2_O_5_) | | CO_2_ | | 0.73 | | Kg CO_2_e kg^-1^ P_2_O_5_ |
|  |  | Potassium (K_2_O) | | CO_2_ | | 0.55 | | Kg CO_2_e kg^-1^ K_2_O |
|  | | | | | | | | |
| Diesel ^2^ | |  | | CO_2_ | | 0.0741 | | Kg CO_2_e MJ^-1^ |
|  | |  | | CH_4_ | | 0.000087 | | Kg CO_2_e MJ^-1^ |
|  | |  | | N_2_O | | 0.00887 | | Kg CO_2_e MJ^-1^ |
|  | | | | | | | | |
| Direct N_2_O from N inputs (synthetic fertilizers) in flooded rice field^2^ | | | | N_2_O | | 6.33 | | Kg CO_2_e kg^-1^ N |
|  |  |  |  |  | |  | |  |
|  | | | | | | | | |
| Indirect losses of N fertilizer in managed soil ^2^ | | Leaching or runoff | | N_2_O | | 1.096 | | Kg CO_2_e kg^-1^ N |
|  |  | Volatilization and re-deposition | | N_2_O | | 0.487 | | Kg CO_2_e kg^-1^ N |

**Table S2.** Farm-level energy use, costs of production, nutrient use, and hired labor for different cropping systems within and outside polders of south-central Bangladesh (±CI). (1 USD = 77.87 BDT; Letters in columns not separated by solid line indicate differences at alpha=0.05 according to the Tukey’s HSD; Significant code ‘***’ 0.001 ‘**’ 0.01 ‘*’ 0.05)

|  | Total Energy Production  (GJ ha^-1^) | Net Energy Yield (GJ ha^-1^) | Total costs of production (USD ha^-1^) | Hired Labor (PSD ha^-1^) | N rate  (kg ha^-1^) | K_2_O rate  (kg ha^-1^) | Wage rate  (BDT PSD^-1^) | *Aman* Price  (BDT kg^-1^) |
| --- | --- | --- | --- | --- | --- | --- | --- | --- |
| a) Within Polders |  |  |  |  |  |  |  |  |
| *Boro –* Fallow *– Aman (BFA)* | 153.95 (9.02) A | 126.76 (8.91) A | 1367.80 (33.88) A | 185.60 (6.70) A | 150.04 (2.12) A | 61.22 (1.51) B | 237.4 (4.4) AB | 20.1 |
| Chili – Fallow *– Aman (CFA)* | 73.24 (6.24) CD | 66.03 (6.16)CD | 1379.34 (23.43) A | 108.64 (4.63) D | 95.80 (1.42) B | 111.89 (1.05) A | 237.8 (3.0) AB | 19.2 |
| Fallow – Fallow *– Aman (FFA)* | 70.40 (1.74) D | 64.89 (1.71) D | 428.97 (6.52) E | 87.88 (1.29) E | 31.18 (0.41) F | 16.45 (0.29) D | 234.6 (0.8) AB | 19.2 |
| Ground nut – Fallow *– Aman (GFA)* | 109.95 (4.51) B | 97.61 (4.46) B | 1073.63 (16.94) B | 133.71 (3.35) B | 48.16 (1.06) C | 37.49 (0.76) C | 237.8 (2.2) A | 19.6 |
| Lathyrus – Fallow – *Aman (LFA)* | 82.25 (2.83) C | 73.80 (2.79) C | 619.44 (10.62) D | 111.02 (2.10) D | 44.60 (0.66) D | 16.23 (0.47) D | 233.0 (1.4) B | 19.0 |
| Mungbean – Fallow – *Aman (MFA)* | 81.00 (1.80) C | 72.81 (1.78) C | 663.06 (6.76) C | 121.49 (1.34) C | 42.73 (0.42) E | 16.49 (0.30) D | 234.6 (0.9) AB | 19.2 |
| *F-value* | 110.86*** | 69.00*** | 2430.62*** | 416.51*** | 3620.41*** | 7151.7*** | 3.76** | NS |
|  | Total Energy Production (GJ ha^-1^) | Net Energy Yield (GJ ha^-1^) | Total costs of production (USD ha^-1^) | Hired Labor (PSD ha^-1^) | N rate  (kg ha^-1^) | K_2_O rate  (kg ha^-1^) | Wage rate  (BDT PSD^-1^) | Aman Price  (BDT kg^-1^) |
| b) Outside polders |  |  |  |  |  |  |  |  |
| *Boro –* Fallow *– Aman (BFA)* | 146.40 (6.63) A | 118.68 (6.51) A | 1355.08 (41.44) A | 183.42 (9.57) A | 153.24 (3.52) A | 60.26 (1.97) A | 228.6 | 18.75 |
| Fallow – Fallow – *Aman (FFA)* | 61.29 (1.91) C | 55.66 (1.88) C | 426.30 (11.96) D | 89.16 (2.76) D | 32.24 (1.02) C | 16.90 (0.57) B | 230.6 | 18.37 |
| Lathyrus – Fallow – *Aman (LFA)* | 72.00 (2.68) B | 63.56 (2.63) B | 605.50 (16.75) C | 110.21 (3.87) C | 44.36 (1.42) B | 16.26 (0.80) B | 231.2 | 18.21 |
| Mungbean – Fallow – *Aman (MFA)* | 71.99 (2.15) B | 63.63 (2.11) B | 654.62 (13.42) B | 122.78 (3.10) B | 44.32 (1.14) B | 17.38 (0.64) B | 230.8 | 18.32 |
| *F-value* | 199.52*** | 113.31*** | 704.44*** | 169.53*** | 1407.97*** | 603.08*** | NS | NS |
